# Electrophysiological Evidence of a Delay in the Visual Recognition Process in Young Children

**DOI:** 10.3389/fnhum.2015.00622

**Published:** 2015-11-20

**Authors:** Catarina I. Barriga-Paulino, Elena I. Rodríguez-Martínez, Mª Ángeles Rojas-Benjumea, Carlos M. Gómez González

**Affiliations:** Human Psychobiology Laboratory, Department of Experimental Psychology, University of SevilleSeville, Spain

**Keywords:** development, Delayed Match-to-Sample, visual short-term memory, contralateral negativity, selection negativity, visual recognition process, matching

## Abstract

The present study analyzes the development of the visual recognition processing of the relevant stimulus in a Delayed Match-To-Sample (DMS) task during the matching phase. To do so, Electroencephalograms of 170 subjects between 6 and 26 years old were recorded. Behavioral responses and Event Related Potentials (ERPs) induced by the stimuli were obtained. Reaction times and errors, mainly omissions, were inversely related to age. The ERPs analysis showed a parietal negativity in the P7 and P8 electrodes when the relevant stimulus was presented in the contralateral site. This negativity resulting from the recognition and selection of the relevant stimulus was present in all age groups. However, the youngest children showed an extended latency in the recognition process. The results suggest that children and adults use similar processes to recognize the item maintained in visual short-term memory (VSTM), but children need more time to successfully recognize the memorized item.

## Introduction

The matching or recognition phase of the visual short-term memory (VSTM) process during Delayed Match-to-Sample (DMS) tests corresponds to the active search of correspondence between the stored item maintained in memory and the presented items. The neural signature for this process has scarcely been studied in children. The present study focuses on the Event Related Potentials (ERPs) associated with the matching process in VSTM, in order to establish whether the same processes are acting in children from 6 years old and in young adults in the recognition of the item maintained from a previously presented visual display.

The stimuli selection on the basis of non-spatial features, such as color or shape, elicits a broad negative ERP termed *Selection Negativity* (SN), which begins between 140 and 180 ms post-stimulus and persists for another 200 ms or more (Hillyard and Anllo-Vento, 1998). SN is thought to reflect the selection of relevant and irrelevant visual stimuli at an early level of information processing (Wijers et al., 1989). SN is elicited in the presence of the selected feature surrounded by distracters' feature; however, the representation of the selected feature must be active to permit the matching between that feature and the item containing it. This type of stimulus selection is assumed to be based on a rapid analysis of the physical features of the stimuli before their properties are fully analyzed. SN is best observed in difference waves, in which the ERP elicited by a stimulus with the unattended feature is subtracted from the ERP elicited by the same stimulus when it has the relevant attended feature. The onset of the SN waveform provides a high-resolution measure of the time at which a particular feature or combination of features is discriminated and selectively processed based on task relevance. In a study conducted by Anllo-Vento et al. (1998), the authors tried to determine the timing and anatomical sources of the SN during attention to color, where subjects (between 19 and 31 years old) attended to either red or blue stimuli. The difference waves formed by subtracting the ERP elicited by a given colored stimulus when it was not attended from the ERP elicited by the same stimulus when it was attended included a prominent SN elicited during the 160–350 ms interval after stimulus onset, with a scalp distribution narrowly focused over the posterior visual cortex.

Another ERP, called N2pc, is related to the visual selection of the stimulus. The deployment of attention to an object in the left visual field (LVF) or the right visual field (RVF) provokes an imbalance in the activity of the contralateral vs. ipsilateral cortical visual areas in the posterior part of the brain. This cortical imbalance can be measured by subtracting the ERPs in the ipsilateral site to the item to be selected from the contralateral site in parietal electrodes. This lateralized component is usually observed between approximately 200 and 300 ms post-stimulus onset, and it is called N2 posterior contralateral or N2pc due to its latency in the N2 time range, negative polarity, and posterior contralateral scalp distribution (Luck and Hillyard, 1994). The N2pc has a clear posterior scalp distribution with a maximum often around electrode PO7 for targets in the RVF, and PO8 for targets in the LVF. Source localization analyses of magnetoencephalographic recordings suggest that the neural generators of the N2pc are in the extrastriate visual cortex with a possible parietal contribution (Hopf et al., 2000). This component is usually measured as the difference in activity at electrode sites PO7 and PO8.

Recent research has revealed a new imbalance in brain activity, similar in latency and morphology to an N2pc, but related to the delayed recall of information in memory (Kuo et al., 2009; Dell'Acqua et al., 2010). In their experiments, these authors presented a memory display containing an equal number of items in the LVF and RVF simultaneously. After a retention period, one item was presented at fixation, and subjects had to indicate if it was present or absent from the initial memory array by pressing a key. This task introduced an imbalance in voltage scalp activity when the centrally-presented probe matched one of the original forms. This imbalance produced a negative difference wave at more anterior electrode sites than for the N2pc, namely at P7-P8 and T3-T4. A similar contralateral negativity to N2pc is also obtained when a single stimulus to be memorized is presented and, after a delay period, the same item is displayed with other items and has to be recognized. In this case, a contralateral negativity to the location where the remembered item is presented appears in posterior areas (Kuo et al., 2009). These findings have led to the hypothesis that at least part of the visual memory trace is likely to be located in the hemisphere contralateral to the hemifield from which the visual information was initially encoded. A study conducted by Shimi et al. (2014), using a visual short term memory task with three different types of trials (pre-cue trial, retro-cue trial, and neutral trial), comparing adults with 10–11 year old children found differences between these two groups with regard to the spatial distribution and latency of some components (Early Directing Attention Negativity, Anterior Directing Attention Negativity, Late Directing Attention Positivity, and N2pc). The N2pc, related to the search for the target item in the memory, appeared earlier, and presented a longer duration in children than in adults.

Therefore, the presence of negativities associated with the selection process, SN and N2pc, in different types of paradigms seems to be well established in humans. Additionally, animal studies have demonstrated that on a VSTM task, inferotemporal neurons sensitive to features of the test stimulus (S2) increase their neural firing in response to the test stimulus when it is coincident with the presented probe (S1); however, a decrease in activity is observed if the stimulus is not coincident with the probe (Chelazzi et al., 1993, 2001). ERPs and single cells both suggest that an increase in neural activity occurs in the contralateral side to the stimulus to be selected. However, no studies have systematically followed the developmental changes in negativities related to the selection process on VSTM tasks, and this is the objective of the present report. Although both SN and N2pc would be suitable for following the selection process, the procedure of subtracting, in a given electrode, the neural activity of a non-target stimulus presented in the contralateral hemifield from the neural activity in the same electrode when a target is presented in the contralateral hemifield has been used. This approach has the advantage of offering a direct comparison with animal studies, and it would allow a direct interpretation.

The aim of the present study is to analyze the ERPs induced by the visual recognition process of an item maintained in VSTM in a developmental study with subjects from 6 to 26 years of age. This approach makes it possible to observe any differences and/or similarities in the neural processes involved in the recognition operation in different age groups from childhood to young adulthood. Several studies have pointed out the rapid changes related to attention in young children. Rebok et al. (1997) and Klenberg et al. (2001) found rapid changes in attention between ages 8 and 10, and these changes became more gradual between ages 10 and 13. In paradigms related to the search for a stimulus' characteristics, Day (1978) showed a decrease in RT from 7 to 12 years old. Lobaugh et al. (1998) showed that children in the 7–8 year-old age range already presented a feature conjunction search similar to adults when the RTs were analyzed. In the case of accuracy, they found similar accuracy to that of adults only in the 11–12 year-old group. The increased variability in errors observed in children between 6 and 8 years old on a visual search task conducted by Rojas-Benjumea et al. (2013) suggested that there is a broad maturational window for the response decision process in young children.

In this study, we hypothesize that in the matching phase of the VSTM task, an SN-like response will emerge during the process of recognizing the contralateral stimulus as a target (S2) because it corresponds to the memorized probe (S1). This negativity will be functionally similar to the SN and present a reduction in SN peak latency with age.

**Figure 1:**
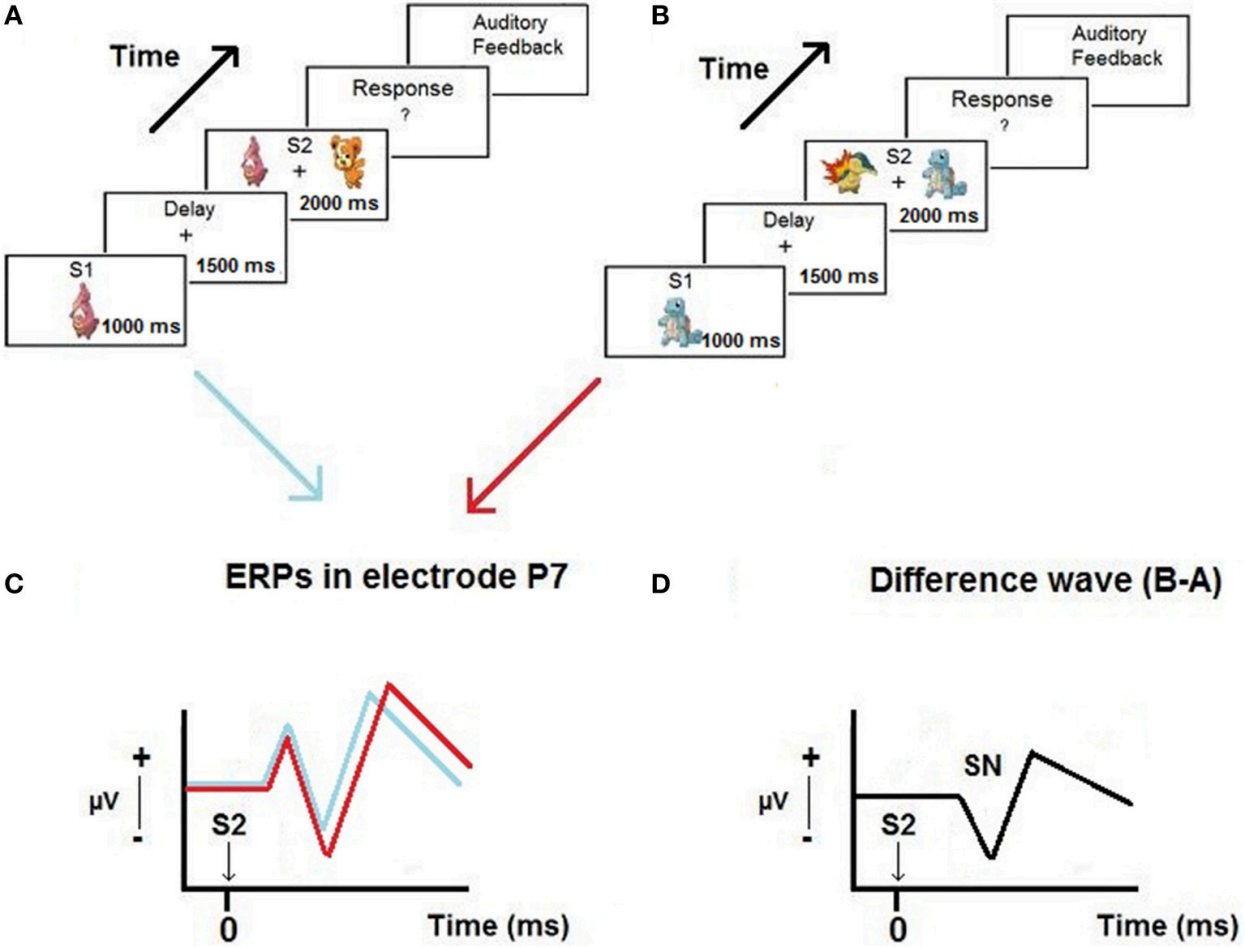
**Example of trials of the Delayed Match-to-Sample paradigm used in the experiment (A,B), ERPs obtained in P7 when the relevant stimulus appear on the left and on the right of the screen (C) and the selection negativity (SN) obtained from the difference waves (D)**.

Figure 1 presents a scheme of the proposed hypothesis.

## Methods

### Subjects

One-hundred and seventy subjects between 6 and 26 years old participated in this study (15.89 years ± 6.12). For each age, eight subjects were recorded and analyzed (4 males and 4 females), with a total sample of 85 males and 85 females. However, two subjects were excluded (a 9-year-old male and a 6-year-old female) due to excessive EEG artifacts. The final sample was composed of 168 subjects, 84 males, and 84 females (16.00 years ± 6.07).

This study included subjects up to 26 years old because we wanted volunteers who had already completed their cerebral maturation. The children who participated were at least 6 years old because we observed that younger children were not capable of performing the entire task. They had difficulty paying attention during the experiment, trouble understanding the instructions, poor attention, and/or fatigue.

Subjects did not report any neurological diseases or psychological impairments, and they were extracted from middle-class socioeconomic backgrounds. The children had good academic records and were recruited from public schools, and the young adults were college students recruited through advertisements on the University Campus.

Experiments were conducted with the informed and written consent of each participant (parents or tutors in the case of children), following the Helsinki protocol. The study was approved by the Ethical Committee of the University of Seville.

### Stimuli and task procedure

Visual stimuli were cartoons of Pokemons and Digimons types. The size of all stimuli was adapted in Picassa to equal dimensions of 142 × 228 pixels. Uncommon stimuli were used to avoid verbal strategies and to ensure that memorization processing was mainly visual. The stimulus presentation program used was E-Prime version 2.0, and a SRBOX Cedrus was used to record the subjects' responses.

The paradigm used was a DMS task composed of a total of 128 trials organized in four experimental blocks with 32 trials each. The trials were counterbalanced; i.e., in half of them the target stimulus appeared on the LVF, and in the other half the target stimulus appeared on the RVF. The order of presentation was totally random, so that each subject performed a unique sequence of trials. The task was kept relatively simple in order to facilitate the testing of the youngest children.

The task started with the appearance of the first stimulus (S1) at the center of the screen. The stimulus covered a visual angle of 4.56° on the horizontal meridian. Figure 1 shows an example of a trial during the task. The stimulus was presented for 1000 ms and had to be memorized by the subject. Then, a blank screen with a fixation point in the center appeared for 1500 ms. During this delay period, the subjects had to maintain the S1 they had previously seen in memory. After that, two stimuli (S2) appeared for 2000 ms (one the same as S1, and the other different), one on the left, and the other on the right side of the screen. Subjects were instructed to press the left button with their left hand or the right button with their right hand if the previously presented S1 appeared on the left or right side of the screen, respectively. Finally, the trial ended with auditory feedback, providing the subject with a different sound depending on whether a correct or an incorrect response was produced. Then, 2000 ms passed until the start of a new trial. A practice session of 10 trials was carried out before the experimental task. Subjects were instructed to relax their facial muscles, keep their eyes focused on the fixation point presented at the center of the screen, and blink as little as possible. With some young children it was necessary to stay with them in the Faraday box and respond together during the practice trials to make sure that they understood the instructions. The instructions to the subjects were: “In the center of the screen a cartoon will appear that you should memorize. Next, two cartoons will appear, one on the left and the other on the right side of the screen. One of them is the same that you memorized before and the other is different. You must press the left button or the right button depending on the side where the memorized cartoon appears. Then, you will hear a sound telling/informing you whether you did ok.”

The duration of the task was approximately 17 min, and rest periods were allowed between blocks.

### EEG recording and analysis

The EEG was recorded in a dim-light and electrically isolated Faraday box. Recordings were made from from 32 scalp sites (Fp1, Fp2, F7, F3, Fz, F4, F8, FC5, FC1, FC2, FC6, M1, T7, C3, Cz, C4, T8, M2, CP5, CP1, CP2, CP6, P7, P3, Pz, P4, P8, POz, O1, Oz, O2), organized according to the 10–20 system and using an Electro-Cap, with four additional electrodes to record ocular movements. The horizontal right and horizontal left electrodes were located at the outer canthus of each eye to record horizontal eye movements, and the vertical superior and vertical inferior were located above and under the left eye to record vertical eye movements. All the scalp electrodes were compared to an average reference, and impedance was maintained below 10 KΩ. Data were recorded in DC at 512 Hz, with a 20,000 amplification gain using a commercial AD acquisition and analysis board (ANT). EEG was recorded in a dimly lit and electrically isolated Faraday box.

EEG preprocessing was performed with EEGLAB and ERPs were obtained in Matlab.

After eliminating the 10 practice trials, the EEG was segmented into 8000 ms epochs, starting 1000 ms before the onset of S1 and lasting until the presentation of the auditory feedback. Only trials with correct responses were analyzed. The EEG was re-referenced to the mastoid average (M1 + M2/2).

Independent Component Analysis (ICA) was applied to the EEG recordings. This method makes it possible to separate the electroencephalographic signal into components in order to correct the influence of artifacts (e.g., blinks, muscle activity, etc.) on the EEG. These components were identified and manually removed based on topographic distribution and frequency criteria. The artifactual components related to blinks showed a frontal location and high power in low frequencies (0–2 Hz). The electromyography signal showed a lateral location around mastoids, but also frontopolar locations, often caused by facial tension, and a high amplitude in frequencies higher than 50 Hz. These components and those with a high power content in 50 Hz were discarded, and the EEG signal was reconstructed from the remaining components. Then, and due to numerous recordings with EEG linear drifts, the EEGLAB function *Linear Detrend* was used to remove the linear trend from the recordings.

A low pass filter of 25 Hz was applied to all the recordings to eliminate the higher frequencies, and in five subjects' EEG recordings a high pass filter of 0.5 Hz was also applied to reduce artifactual slow waves, once the EEG recordings of these specific subjects showed more noise in the slow brain activity than in the EEG recordings of the other subjects. Finally, an artifact rejection protocol was applied, discarding from the average the recorded voltages that exceeded ±100 μV in the recordings of subjects from 16 years old on, and ±150 μV in the recordings of subjects up to 15 years old in any channel, in order to eliminate any extra-cerebral contamination. Distinct voltage values were applied due to the differences commonly observed in the spectral power of children's and adults' recordings. Children present a higher spectral power than adults do (see, for example, Barriga-Paulino et al., 2011). In the case of having applied ±100 μV to all subjects, which is usually the standard value applied in the artifact rejection, many non-artifactual trials of the children's recordings would have been eliminated from the electrophysiological data. The ERPs obtained from children presented a quite similar morphology and noise level to those of adults, indicating that the procedure of selecting different voltage windows for artifact rejection in different ages was appropriate and did not distort the results in the inter-group comparisons.

To analyze the object's matching phase, the baseline was between 0 and 100 ms before the S2 onset, corresponding to the 2400–2500 ms period of the entire trial. Then, subjects were averaged for each condition, LVF and RVF target presentation, and the sample was divided into 5 age groups (children, 6–9; pre-adolescents, 10–13; adolescents, 14–17; emergent adults, 18–21; and young adults, 22–26 years old). The difference wave between the two conditions was calculated (target in RVF minus target in LVF and target in LVF minus RVF), and the ERPs were filtered above 7 Hz to avoid high frequency noise. The difference waveforms corresponding to the RVF target stimulus presentation minus the LVF target stimulus presentation were represented in P7, and the waveforms obtained from the opposite subtraction were represented in P8. The age group from 6 to 9 years old was also sub-divided into two groups (6–7 and 8–9 years old) in order to analyze whether there were differences at these earlier ages. This age window is particularly characterized by fast developmental changes in attention, as mentioned in the introduction, and so it is important to closely analyze possible differences at these specific ages.

The difference waveforms were also represented in P7 and P8, respectively, for these age groups, together with the group from 10 to 13 years old, in order to compare the ERPs of the 3 youngest age groups.

Topographical maps for voltage and Current Source Density (CSD) were obtained for two temporal windows, 150–250 ms and 350–450 ms (the first one for the 5 age groups, and the second for the 3 youngest groups), using the BESA software (Brain Electrical Source Analysis).

### Statistical analysis

Regarding the behavioral data analysis, the means of the reaction times and the percentages of the three types of errors (commissions, omissions, and anticipations) and their standard deviations were calculated. Anticipation was defined as a response to S1, a response <200 ms after the S2 appearance, and/or a response in the interval between S1 and S2. Regressions between age and the behavioral measures were obtained.

To analyze possible statistically significant differences in the ERPs among the age groups (for the 5 and 3 age group sub-divisions), three latency windows were defined, 150–250 ms, 250–350 ms, and 350–450 ms, and the mean amplitudes of the ERPs were analyzed. A Mixed-Model Repeated-Measures ANOVA was computed, with the visual hemifield in which the target stimulus was presented (LVF and RVF), hemisphere (left and right), and pairs of electrodes (P3/P4, P7/P8, and O1/O2) as within-subjects factors, and the age groups as between-subject factor. The Greenhouse-Geisser correction was used to correct sphericity.

Of all the possible interactions, the interaction Hemisphere × Visual Hemifield × Age Group was further explored in order to analyze whether the contralateral negativity component (SN) changed its amplitude in the different age groups. The electrodes, visual field of stimulation, and hemisphere were collapsed. The following equation was used to obtain the contralateral negativity to compare the amplitude of the age groups:

$$\begin{array}{l} \text{CN}=\left[\left(\text{P}7+\text{P}3+\text{O}1\right)\text{RVF}-\left(\text{P}7+\text{P}3+\text{O}1\right)\text{LVF}\right]\\ +\left[\left(\text{P}8+\text{P}4+\text{O}2\right)\text{LVF}-\left(\text{P}8+\text{P}4+\text{O}2\right)\text{RVF}\right]∕2 \end{array}$$

To compute the peak latency of the SN, we obtained the time at which the minimum value (most negative value) appeared in electrodes P7 (difference wave between RVF minus LVF target presentation) and P8 (difference wave between LVF minus RVF target presentation). To obtain the minimum value of the difference wave, the “min” function of Matlab was applied in the time window 150–450 ms. These values were regressed against age, using an inverse model. In addition, the RTs were regressed against the SN peak latency in the P7 and P8 electrodes, using a linear model. In order to evaluate the relative contribution of SN and age to the RTs, the multiple regressions RT = c1 ^*^ P7 + c2 ^*^ Age and RT = c1 ^*^ P8 + c2 ^*^ Age were computed.

## Results

### Behavioral data

Omissions were the most common type of error performed by children, making up more than 50% of total errors (mean percentage ± SD of incorrect responses, 1.11 ± 4.32; mean percentage ± SD of anticipations, 0.50 ± 1.52; mean percentage ± SD of omissions, 2.03 ± 3.99). The inverse regression of the different behavioral parameters with age was statistically significant (RTs: *R*^2^ = 0.546, *p* < 0.001; Incorrect responses: *R*^2^ = 0.076, *p* = 0.005; Anticipations: *R*^2^ = 0.160, *p* < 0.001; Omissions: *R*^2^ = 0.416, *p* < 0.001), indicating a decrease in the reaction times and in the three types of errors with age.

Table 1 shows the means and standard deviations of the reaction times and the percentage of errors for each age group.

**Table 1:** **Means of reaction times and standard deviations for responses to the S2, and percentage of each type of error for each age group**.

**Age group**	**Mean of RTs**	**Standard deviation of RTs**	**Total errors**	**Incorrect responses**	**Anticipations**	**Omissions**
6–9	846 ms	172.28	7.79	2.47	0.81	4.52
10–13	643 ms	148.47	2.39	0.61	0.51	1.27
14–17	553 ms	116.75	0.73	0.46	0.07	0.20
18–21	466 ms	89.95	0.51	0.44	0.05	0.02
22–26	517 ms	145.63	0.39	0.33	0.02	0.04

### Event related potentials

**Figure 2:**
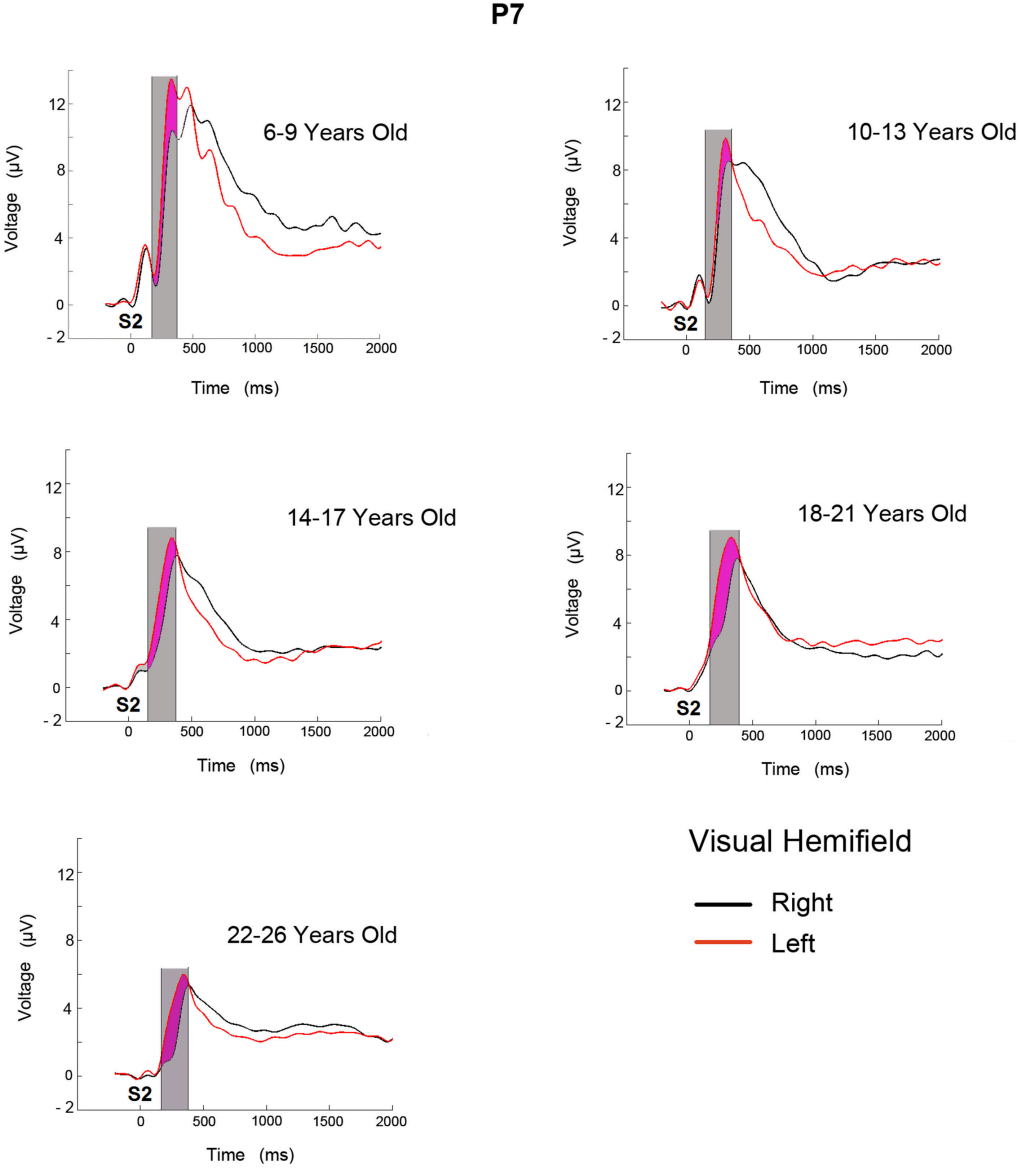
**ERPs obtained in electrode P7 from LVF and RVF stimulation for five age groups**. The time at which the second stimulus appears is labeled as S2. The area shaded in gray corresponds to the analyzed temporal window and the purple area corresponds to the increased negativity when target is presented in the contralateral side.

Figure 2 shows the ERPs for the two conditions (target stimulus in the LVF and RVF target presentation) in the P7 electrode for the 5 age groups. In the displayed P7 electrode, the successive ERP components for all age groups seem to be modulated by a negative wave when the target stimulus appears in the RVF (contralateral to the recording site). In the 6–9 year old age group, the ERP modulation extends for a longer duration. The analyzed temporal window is shaded in gray and the negativity obtained from the subtraction of the two conditions is shaded in purple.

**Figure 3:**
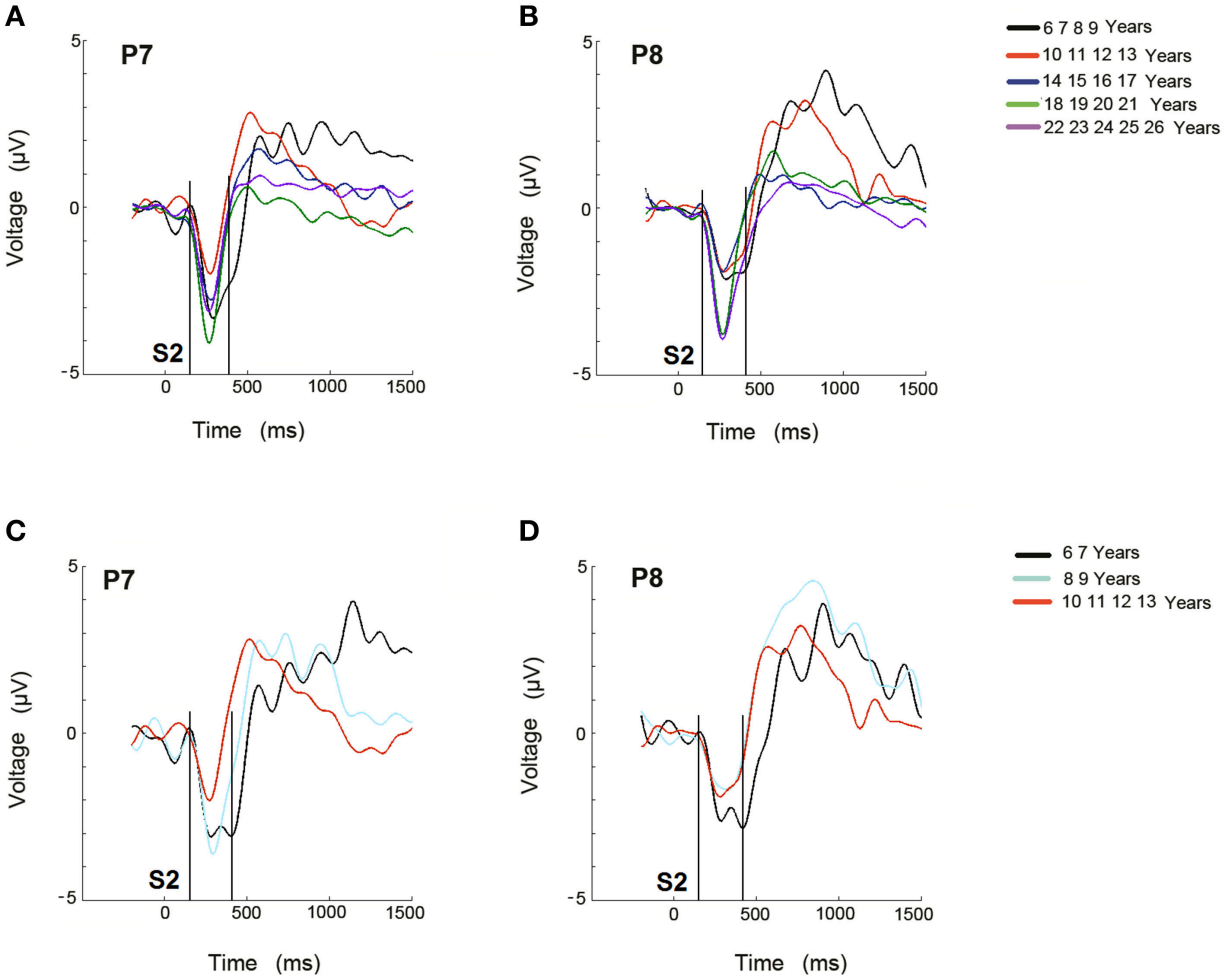
**Difference waveforms of ERPs obtained from RVF stimulation minus LVF stimulation for the 5 age groups (A) and for the 3 youngest age groups (C) in electrode P7 during the recognition of the target stimulus**. Difference waveforms of ERPs obtained from LVF stimulation minus RVF stimulation for the 5 age groups **(B)** and for the 3 youngest age groups **(D)** in electrode P8 during the recognition of the target stimulus. The time at which the second stimulus appears is labeled as S2. The area where the Selection Negativity appears and is statistically analyzed is labeled as SN.

The ERP difference waves, RVF minus LVF and vice versa, are represented in the P7 and P8 electrodes, respectively, for the 5 age groups (Figures 3A,B), and for the 6–7, 8–9, and 10–13 year old groups in Figures 3C,D.

A qualitative description indicates that in the 6–9 year old group, the negativity (labeled as SN) lasted longer than in the other age groups (Figure 3A). A similar delay appears for the negativity in the P8 electrode (Figure 3B). In the figures corresponding to the sub-division of the youngest group into a 6–7 year old group and an 8–9 year old group, along with the 10–13 year old group, the 6–7 year old group also presented an extended duration of this negativity in both hemispheres (Figures 3C,D).

### Current source density maps

**Figure 4:**
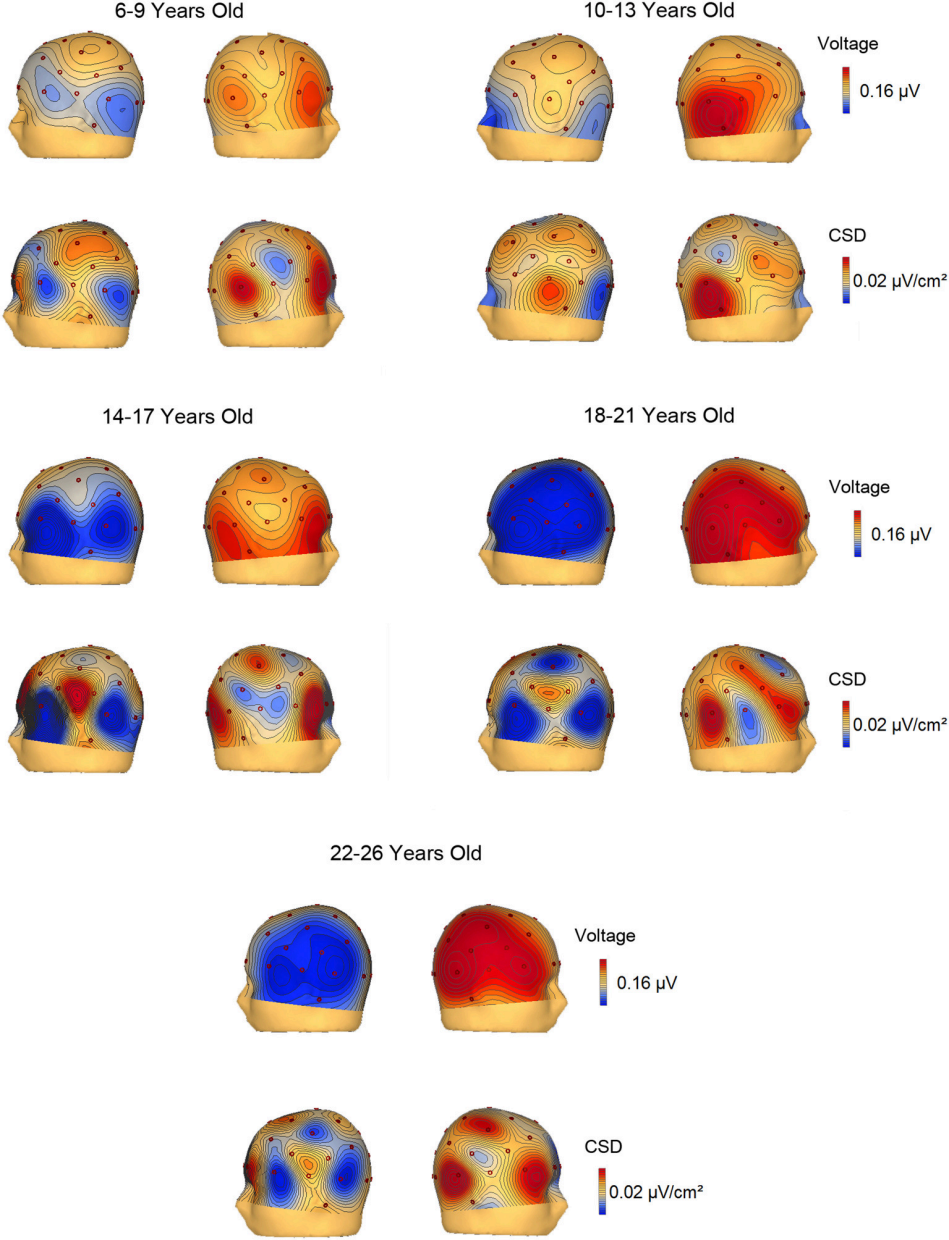
**Voltage and current source density maps for the 5 groups in the temporal window 150–250 ms after the S2 onset**.

**Figure 5:**
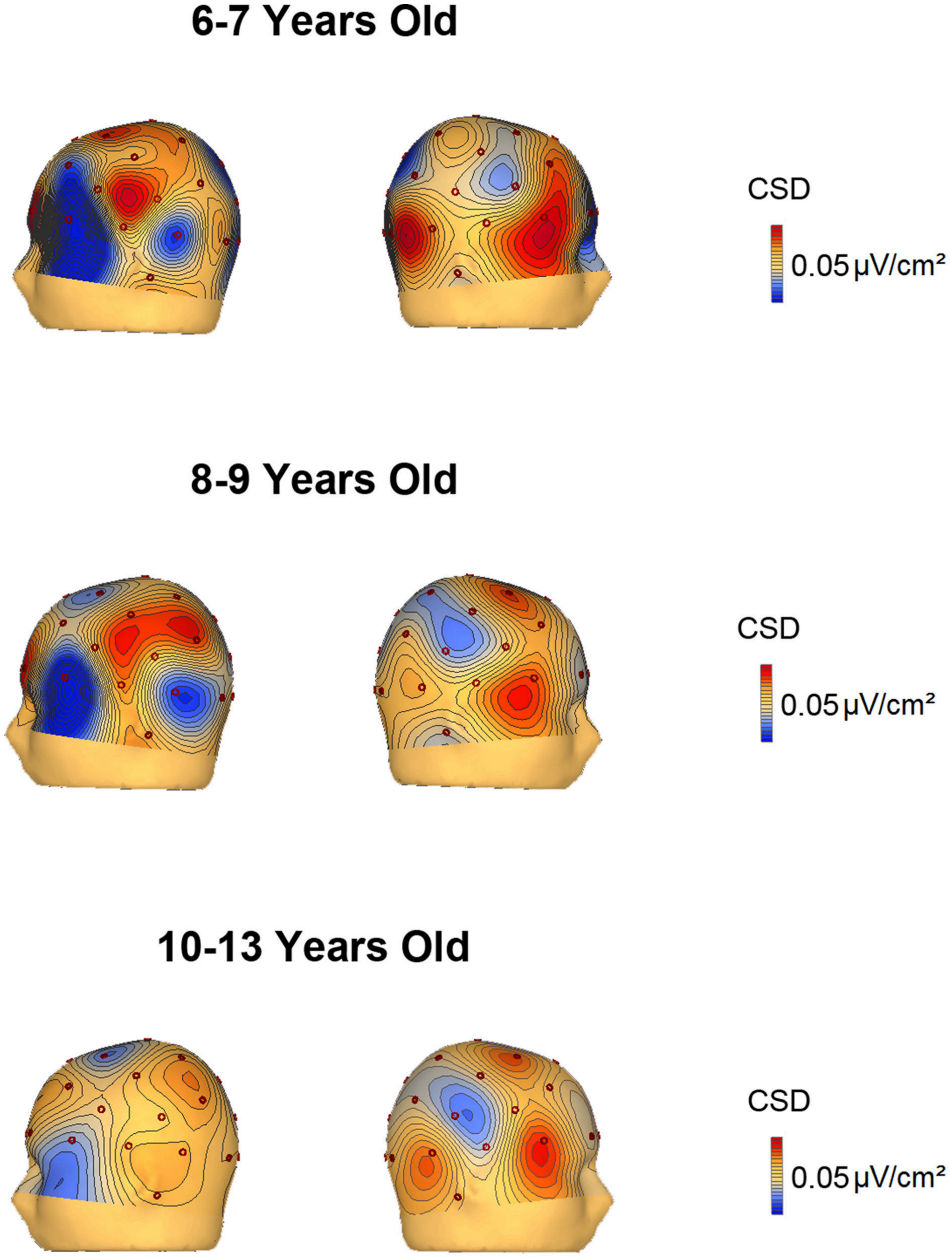
**Voltage and current source density maps for the 3 youngest groups in the temporal window 350–450 ms after the S2 onset**.

The voltage and CSD maps of the ERP difference waves (RVF minus LVF) were performed in BESA in two temporal windows, 150–250 ms (Figure 4) for all age groups and 350–450 ms (Figure 5) for the 3 youngest groups (6–7, 8–9, 10–13 years old). The CSD maps are presented in order to show that the anterior and posterior foci correspond to two distinct electrical activities.

In the voltage maps of the 150–250 ms time window, a negative focus in the parietal region of the left hemisphere can be observed in the two youngest groups (6–9 and 10–13 year old groups). The CSD maps made it possible to separate a left anterior current sink, probably corresponding to eye movements (and the corresponding right hemisphere anterior current source), from the posterior left parietal current sink and right parietal current source.

The CSD topography in the 350–450 ms time window (Figure 5) was also represented for the 6–7, 8–9, and 10–13 year old groups, given that the contralateral to the target negativity extended longer in time in the young children compared to the other age groups. In the 350–450 ms temporal window, it can be observed that up to 9 years old, the parietal negativity is present in the left hemisphere (and mirrors positivity in the right hemisphere). This result indicates that the youngest children extended the processing indexed by the contralateral to the target negativity for a longer period than the 10–13 year old subjects.

### Statistical analysis

Mixed ANOVAs of Hemisphere × Visual Hemifield × Electrodes × Age Group were computed on the ERPs induced by the target stimuli for the three considered time windows. Supplementary Figure 1 shows the mean voltage in the left hemisphere and the right hemisphere (collapsing electrodes) when stimuli are presented on the LVF and on the RVF in the three time windows. Data in Supplementary Figure 1 show the increased negativity when targets are presented in the contralateral hemifield to the recorded electrodes, suggesting a Hemisphere × Visual Hemifield interaction.

Although all the statistically significant effects are reported, only those in which the Hemisphere × Visual Hemifield × Age Group interaction was significant are further explored, given that the primary interest in the present report is the SN in the matching process during development. Table 2 reports all the ANOVA results.

**Table 2:** **ANOVA repeated measures with five age groups as inter-group factor and three within-subjects factors: Hemisphere (left, right), Visual Hemifield of target presentation (left, right), and Electrodes (P3, P4, P7, P8, O1, O2)**.

	**ANOVA repeated measures**			
	**150–250 ms (five age groups)**	**250–350 ms (five age groups)**	**350–450 ms (five age groups)**	**350–450 ms (three youngest groups)**
Group effect	No significant	Significant effects of the group [*F*_(4, 163)_ = 12.176, *p* < 0.001]	Significant effects of the group [*F*_(4, 163)_ = 8.921, *p* < 0.001]	Significant effects of the group [*F*_(2, 93)_ = 6.336, *p* = 0.003]
Principal effects	No significant	Hemisphere [*F*_(1, 163)_ = 41.563, *p* < 0.001]	Hemisphere [*F*_(1, 163)_ = 31.941, *p* < 0.001]	No significant
Significant interactions	Hemisphere × Group [*F*_(4, 163)_ = 2.589, *p* = 0.039] Hemisphere × Visual hemifield [*F*_(1, 163)_ = 133.046, *p* < 0.001] Hemisphere × Visual Hemifield × Age group [*F*_(4, 163)_ = 5.579, *p* < 0.001]	Hemisphere × Visual hemifield [*F*_(4, 163)_ = 224.207, *p* < 0.001] Hemisphere × Visual hemifield × Age group [*F*_(4, 163)_ = 4.077, *p* = 0.004]	Hemisphere × Visual hemifield [*F*_(4, 163)_ = 3.880, *p* = 0.051] Hemisphere × Visual hemifield × Age group [*F*_(4, 163)_ = 2.858, *p* = 0.025]	Hemisphere × Visual hemifield interaction [*F*_(1, 93)_ = 5.962, *p* = 0.018] Hemisphere × Visual hemifield × Age group [*F*_(2, 93)_ = 5.468, *p* = 0.007]

*ANOVAs were computed independently for three temporal windows (150–250, 250–350, 350–450 ms). In the last temporal window an additional ANOVA was computed for the three youngest groups*.

#### Temporal window 150−250 ms

There were no main effects of the group, the hemisphere or the stimulated visual field. The interaction of the effects of Hemisphere × Visual Hemifield × Age Group was further explored. To analyze this interaction, focusing on differences in the age group, the electrode, visual field, and hemisphere factors were collapsed, as indicated in the methods section, and the differences in the contralateral negativity in the different age groups were compared. The Bonferroni comparisons indicated that children presented a statistically lower negativity contralateral to the target than the young adult and adult groups (*p* = 0.012, *p* = 0.049).

**Figure 6:**
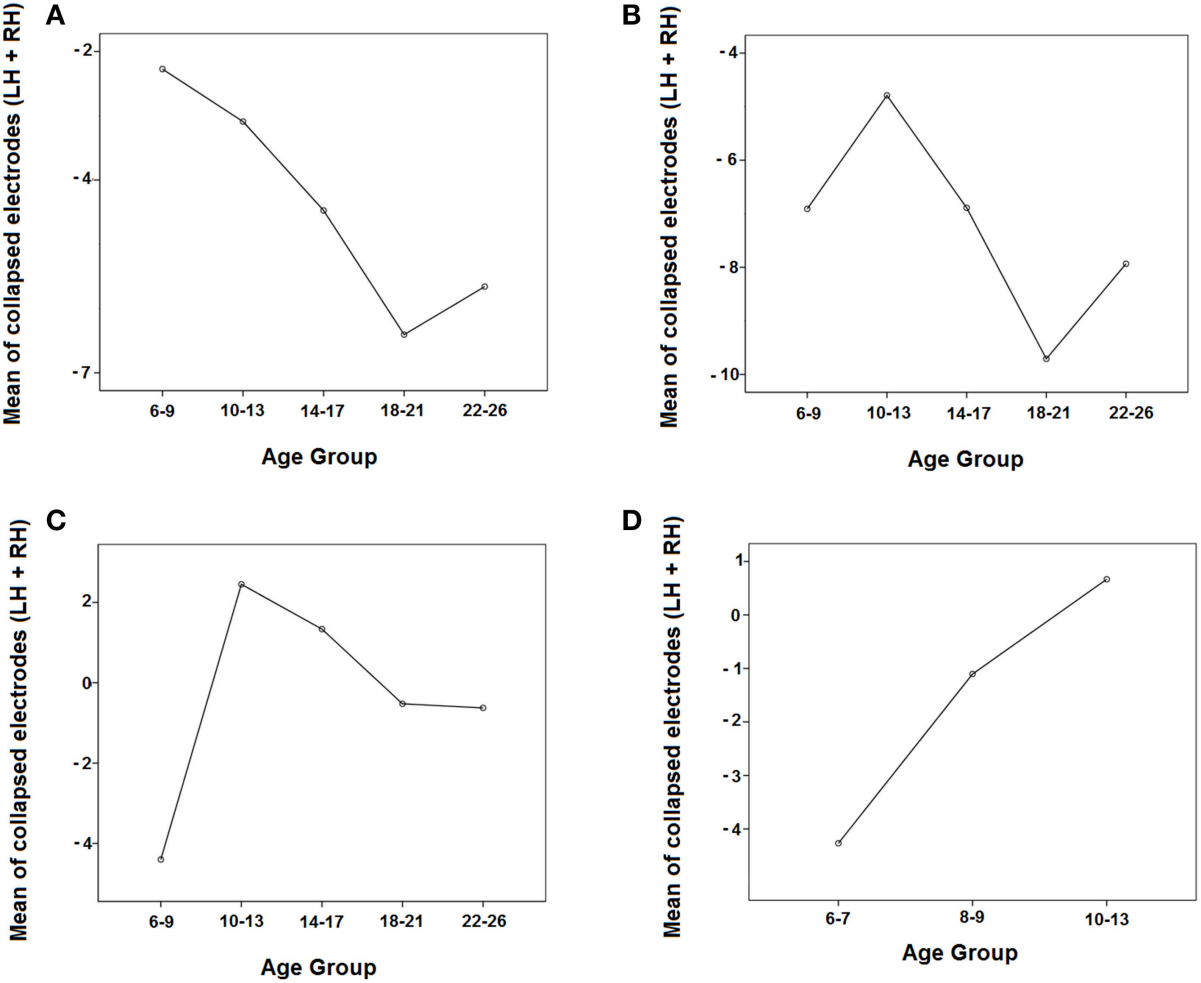
**Evolution of the negativity contralateral to the visual hemifield in the 5 age groups in the three time windows (A: 150–250 ms, B: 250–350 ms, C: 350–450 ms) and in the three youngest groups in the 350–450 ms time window (D) by collapsing the parieto-occipital electrodes of both hemispheres (P3, P7, O1 and P4, P8, O2)**. LH, Left Hemisphere; RH, Right Hemisphere.

Figure 6A shows that the negativity contralateral to the visual hemifield increased with age.

#### Temporal window 250−350 ms

In the middle temporal window, there were statistically significant effects of the group, hemisphere, and the interactions Hemisphere × Visual Hemifield and Hemisphere × Visual Hemifield × Age Group (Table 2). Supplementary Figure 1 indicated that the ERPs in the right hemisphere are more positive in all age groups compared to the left hemisphere, and the ERPs are more negative in the contralateral side to target presentation than in the ipsilateral side. In order to explore the triple interaction (Hemisphere × Visual Hemifield × Age Group), the Bonferroni comparisons between age groups were performed, showing no statistically significant differences among them. Figure 6B shows that the negativity contralateral to the visual hemifield decreased with age and then increased.

#### Temporal window 350−450 ms

In the late temporal window, there were statistically significant effects of the group, hemisphere, and the interactions Hemisphere × Visual Hemifield and Hemisphere × Visual Hemifield × Age Group (Table 2). Supplementary Figure 1 indicated that ERPs in the right hemisphere are more positive in all age groups compared to the left hemisphere, and it can be observed that the youngest group is the only one where the Hemisphere × Visual Hemifield interaction persists. Exploring the Hemisphere × Visual Hemifield × Age Group interaction, the corrected Bonferroni comparisons indicated that children presented a statistically higher negativity contralateral to the target than the preadolescent group (*p* = 0.010). Figure 6C shows that the amplitude of the negativity contralateral to the visual hemifield decreased in preadolescents and then increased from adolescents to adults.

#### Temporal window 350−450 ms for the youngest age groups

As Figures 3C,D suggest that young children presented a contralateral SN that extended longer than that of the other groups, an ANOVA (Hemisphere × Visual Hemifield × Electrodes) was independently computed in the groups from 6 to 7 and 8 to 9 years old in the 350–450 ms time window. The results only showed a significant Hemisphere × Visual Hemifield interaction in the 6–7 year old group [*F*_(1, 15)_ = 9.752, *p* = 0.007]. The 8–9 year old group did not show a significant interaction, indicating that the negativity contralateral to the visual hemifield where the target appears was only present in the 6–7 year old group for the 350–450 ms time window.

Additionally, the omnibus ANOVA (Hemisphere × Visual Hemifield × Electrodes × Age group) was also computed for the three youngest age groups (6–7, 8–9, 10–13 years old).

The ANOVA for the 350–450 ms time window in the three youngest groups showed significant effects of the group and the interactions Hemisphere × Visual Hemifield and Hemisphere × Visual Hemifield × Age Group. Analyzing this last interaction more in-depth, the corrected Bonferroni comparisons indicated that 6–7 year old children presented a statistically higher negativity contralateral to the target than the 10–13 year old group (*p* = 0.005). Figure 6D shows that the negativity contralateral to the visual hemifield decreased with age.

### Regression analysis

**Figure 7:**
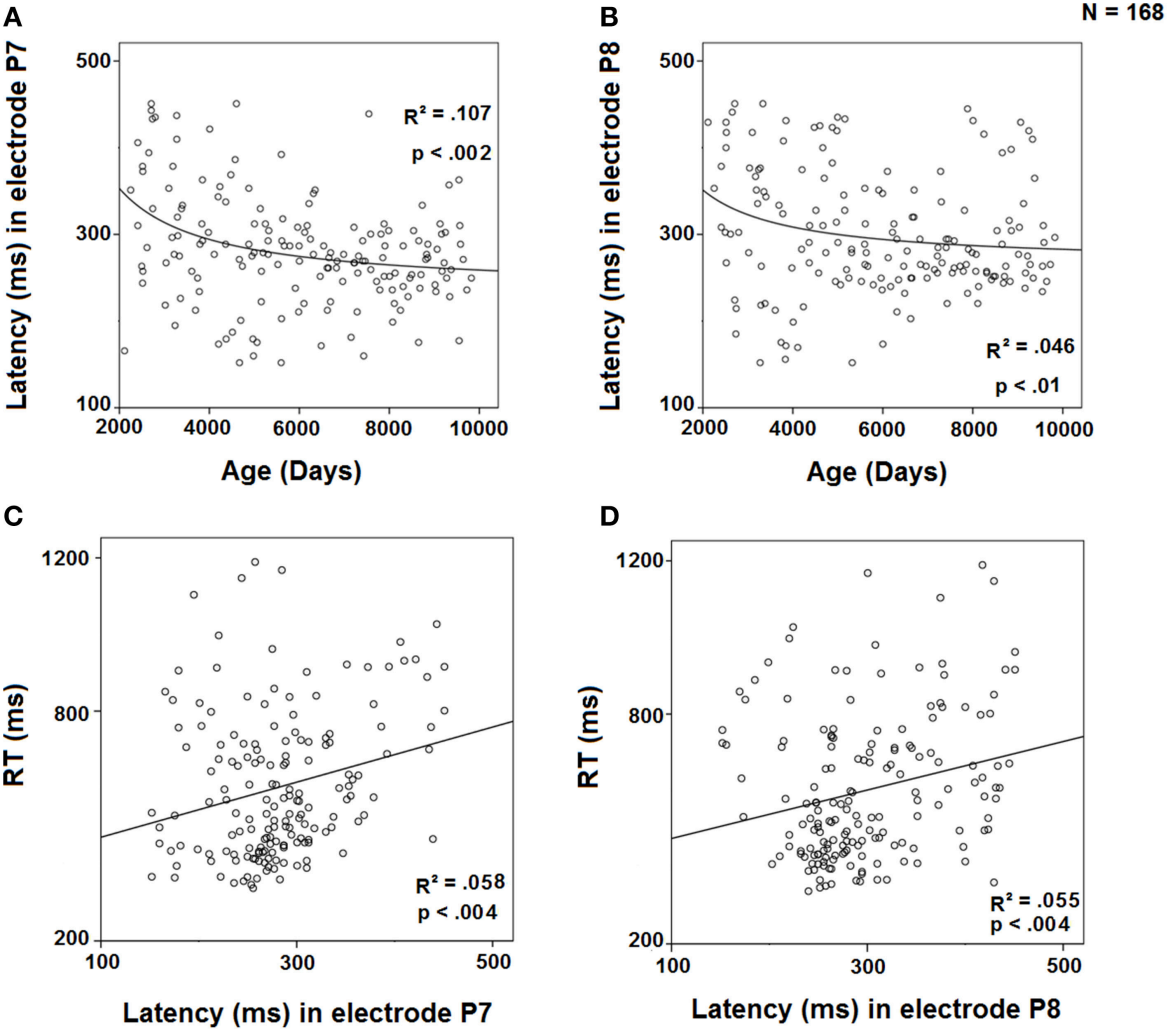
**Regression of the latency of the difference wave in electrode P7 by age (A) and by RT (C) and in electrode P8 by age (B) and by RT (D)**.

The peak latency of the difference wave in the contralateral to the target electrodes (P7 and P8) was regressed against age using an inverse model (Figures 7A,B). The results showed a significant inverse regression for both electrodes, P7 and P8, indicating a decrease in SN latency with age. On the other hand, the regressions of RTs against the peak latency of SN in P7 and P8 (Figures 7C,D) were fitted to a linear model, showing an increase in RTs with the increase in peak latency in those electrodes. The multiple regression RT = c1 ^*^ P7 + c2 ^*^ Age showed that for the P7 electrode (*r*^2^ = 0.403), RT is only explained by age (*p* < 0.001) and not predicted by P7 latency (*p* = 0.297), suggesting that the ability of P7 peak latency to predict RTs is completely mediated by age. However, the multiple regression RT = c1 ^*^ P8 + c2 ^*^ Age showed that for the P8 electrode (*r*^2^ = 0.417), RT is not only explained by age (*p* < 0.001), but also independently by P8 latency (*p* = 0.025).

## Discussion

The goal of the present study was to analyze the visual recognition process during the recognition (or matching) during a DMS paradigm during child to adult development. The subjects had to match one of the visual stimuli presented with the stimulus stored in VSTM. The results showed a contralateral parietal negativity induced by the S2 onset. The negativity was obtained in the difference waves of ERPs induced by displays in which the recognized target stimulus appeared in the RVF minus the ERPs induced when the target stimulus appeared in the LVF (in the P7 electrode). The mirror right parietal positivity (in the electrode P8) was also obtained, although it would correspond to a negativity if the results of subtraction LVF-RVF are observed in the posterior right hemisphere, and then the corresponding mirror positivity appeared in P7. The contralateral negativity to the target presented a tendency to increase with age in early latencies, it lasted longer in children, and it presented a peak latency that decreases with age. The results suggest that children, adolescents, and young adults use the same mechanism (the contralateral parietal negativity) to identify and select the recognized target, although the children spent more time on this process, possibly due to normal neurocognitive immaturity.

This negative contralateral potential would be related to an SN component, an endogenous attention-related negativity that emerges in a stage of selective attention and information processing (Hillyard and Anllo-Vento, 1998). The increase in this posterior negativity for contralateral target stimuli was probably related to the identification and categorization of the stimulus, which frequently implies a comparison with representations stored in memory of visual characteristics that define the target stimulus (Eimer, 1994). In fact, a sustained posterior slow negativity has been proposed as the sharing mechanism in children, adolescents and young adults for retaining the presented item during the delay period of the DMS task (Barriga-Paulino et al., 2014). The parietal negativity observed in the present study, with peak latencies displayed in Figure 7, would be related to the selection or matching process of the visual features that characterize the relevant stimulus stored in the VSTM. Hillyard and Anllo-Vento (1998) found that the selection of the relevant feature was indexed by a broad SN that extended between 150 and 300 ms, followed by a later positivity. Although the experimental paradigm of the present paper is used to analyze working memory and not selective attention as the experimental paradigm used by Hillyard and Anllo-Vento (1998), the contralateral negativity observed during the matching of the stimulus retained in memory with the stimuli presented, consists of a negativity related to the stimulus recognition. In spite of this negativity had been obtained in a different paradigm, presents similar characteristics to the “classical” attentional-related SN, such as cognitive demands (selection), topography, latency, and polarity.

In adolescents between 14 and 17 years old, the coincidence with the maturation of SN in the target-processing waveforms emphasizes the development of selective attention abilities (Oades et al., 1997). In our study, maturation with regard to the selection of the relevant stimulus was observed around 10 years old, the age at which the parietal negativity duration tended to be similar to an adult's. At this age, the peak latency tended to stabilize, and the duration of the SN showed adult values. The developmental study of selective attention to color conducted by van der Stelt et al. (1998), in which the participants were children from 7 years old to adults up to 24 years old, the authors observed an SN as an occipito-temporo-parietally distributed negativity in the 150–300 ms latency range, which was most clearly visible for the 19–24 and 16–18 year old subjects, and to a lesser extent for the 13–15 and 10–12 year old subjects. This ERP presented an increase in amplitude in the older subjects. According to these authors, the fact that SN amplitude increased with age represents neurophysiological evidence that the efficiency of visual selective processes increases during childhood and adolescence. These results indicate that selective mechanisms are operating not only in adults, but also in children, during attentional tasks. Jonkman et al. (2004), in a study comparing ADHD children with controls (between 7 and 13 years old) on an early selective attention task in the visual modality, found an occipital SN at the Oz electrode from 200 to 280 ms in both groups. Ortega et al. (2013) reported reduced SN in ADHD children. However, whether or not similar mechanisms are operating during the recognition phase of WM remains to be explored.

The negativity contralateral to the target could be considered the macroscopic expression of the results obtained by Chelazzi et al. (1993, 2001) showing an increased activity in feature-sensitive neurons when the contralateral stimulus corresponded to a previously presented probe compared to when the contralateral stimulus did not correspond to the presented probe. The authors concluded that this increase in activity was related to the selection process of the contralateral stimulus as the target, allowing the movement to be initiated. At a macroscopic level, although differences in the experimental paradigms appear, they would correspond to the subtraction of target contralateral minus non target contralateral performed in the present report. Similarly, as already suggested in Barriga-Paulino et al. (2014), the negative slow wave obtained during the maintenance phase would be the macroscopic index of the sustained neural activities obtained by Fuster and Jervey (1982) during the maintenance phase in inferotemporal neurons in DMS experiments.

Therefore, the SN obtained in the present report would be a macroscopic index of the increased neural activity when the target is presented in the contralateral side.

Comparing children with the other age groups, results showed that the negativity lasted for a longer time, and the peak latency was higher in children than in adults, as obtained by Shimi et al. (2014). These results indicated that the youngest children showed a delay in the recognition process of the relevant stimulus, taking more time to identify it. In the present experiment, the statistically significant Hemisphere × Visual Hemifield interaction, observed only in the youngest group (6–9 years old) in the 350–450 ms time window, confirms that this age group required more time to process the relevant stimulus than older subjects. When the 6–9 year old group was divided into two age groups (6–7 and 8–9), the ERPs showed that the 6–7 year old group needed more time to process the target stimulus, once the parietal negativity had extended for a longer time than in the other two age groups (8–9 and 10–13 years old). This contralateral parietal negativity was higher in the older groups in the earlier and intermediate time windows (150–250 and 250–350 ms), while in the later time window (350–450 ms), the youngest group showed a higher negativity. This outcome may also be related to the fact that the older children seem more able to quickly exclude or filter out irrelevant information than the younger children. The acquisition of this skill of focusing on or excluding unwanted information may characterize developmental changes in selective attention in different age groups (Pick et al., 1972; Ridderinkhof and van der Stelt, 2000). The regression analysis of the peak latency of the difference wave in the parietal contralateral electrodes (P7 and P8) to the target side with age was inverse, also corroborating that younger subjects were slower to process the relevant stimulus than older subjects.

The CSD and voltage topographical maps provide convergent evidence that the main difference between children and young adults is basically the delay in the identification of the relevant stimulus by the youngest participants. With the objective of localizing the sources and sinks of the lateralized activity during the recognition process, two foci emerged: the expected parietal negativity in the contralateral hemisphere with regard to the visual hemifield where the relevant stimulus was presented, and an ipsilateral positivity in the homologous area. However, a contralateral and anterior current sink and homologous ipsilateral anterior current source were also obtained. These anterior current sink and source were interpreted as corresponding to ocular activity due to inverse polarity and localization. The youngest group (6–9 years old) prolonged the contralateral parietal negativity in the 350–450 ms time range, suggesting a slower recognition process compared to older age groups. However, the topography was not different in any age group, suggesting that the same brain areas are operating in the identification and selection process from 6 years old on.

Comparing the voltage values of both hemispheres, it can be observed a lateralization to the right of the ERPs before any subtraction operation. This hemisphere showed consistently higher voltages than the left hemisphere, a result that could be due to the nature of the stimuli used in the task: cartoons. This lateralization suggests a special role for the right hemisphere for processing these complicated Digimons and Pokemons shapes, as previously described for face processing (Rossion et al., 2000). Although the task was merely visual and we tried to select less known cartoons in order to guarantee that memorization was only made with visual strategies, we have not sure if some subjects (principally children) knew their names and, thus, could use verbal strategies to aid in stimuli memorization. Thus, this aspect consists of a limitation of the study. However, the fact that the right hemisphere presents higher voltages than the left hemisphere in the visually induced ERPs is an indicator that probably this limitation did not affect so much the present results and possibly subjects used strategies mainly visual and not verbal. On the other hand is interesting to notice that cartoons produced increased activity in the right hemisphere for all age groups. The right hemisphere lateralization of ERPs is probably the reason for the less consistent results when the N2pc approach (Luck and Hillyard, 1994; subtracting activity from ipsilateral electrodes to contralateral electrodes) was exploratorily checked (data not shown) instead of the SN approach (subtracting ERPs of LVF stimulation from ERPs of RVF stimulation, and vice versa): ERPs in the right hemisphere presented higher amplitude than ERPs in the left hemisphere, in contrast to obtaining negativity when P8 minus P7 activity is computed during LVF stimulation.

In summary, a contralateral parietal negativity was found with regard to the visual hemifield where the relevant stimulus was presented. This recognition negativity would be similar to the SN, defined as the effort to select the correct feature among irrelevant ones. The youngest children were the subjects that needed the most time to process the correct stimulus, given the extended duration of the negativity compared to the older individuals. The similar topography in different age groups suggests not only the same mechanism, but also that similar brain resources are used in the different age groups in the process of identification and selection of a visual item from VSTM. In conclusion, we obtained similar results to those previously found in several studies about contralateral negativities related to stimuli selection, but with the novelty of analyzing an extended sample comprising ages between 6 and 26 years old and being in the context of a working memory task. In this way, we think the present study provides additional information about the continuous development with age of stimuli identification and selection during VSTM tasks.

## Author contributions

CB: Experiment design, recording, signal processing, statistical analysis, discussion, and manuscript's writing. ER: Experiment design, statistical analysis, and discussion. MB: Experiment design, statistical analysis, and discussion. CG: Experiment design, statistical analysis, and discussion.

### Conflict of interest statement

The authors declare that the research was conducted in the absence of any commercial or financial relationships that could be construed as a potential conflict of interest.

## Supplementary material

Supplementary Figure 1 **Hemisphere × Visual Hemifield interactions in the 150–250, 250–350, and 350-450 ms for all 5 age groups**. In the last time window, the interactions for the sub-groups from 6 to 7 and 8 to 9 years old are also displayed.
