# Supplementary figures and images for: Electrophysiological Evidence of a Delay in the Visual Recognition Process in Young Children
Source: Front Hum Neurosci. 2015 Nov 20;9:622. doi: 10.3389/fnhum.2015.00622 (PMC4653287; doi:10.3389/fnhum.2015.00622)

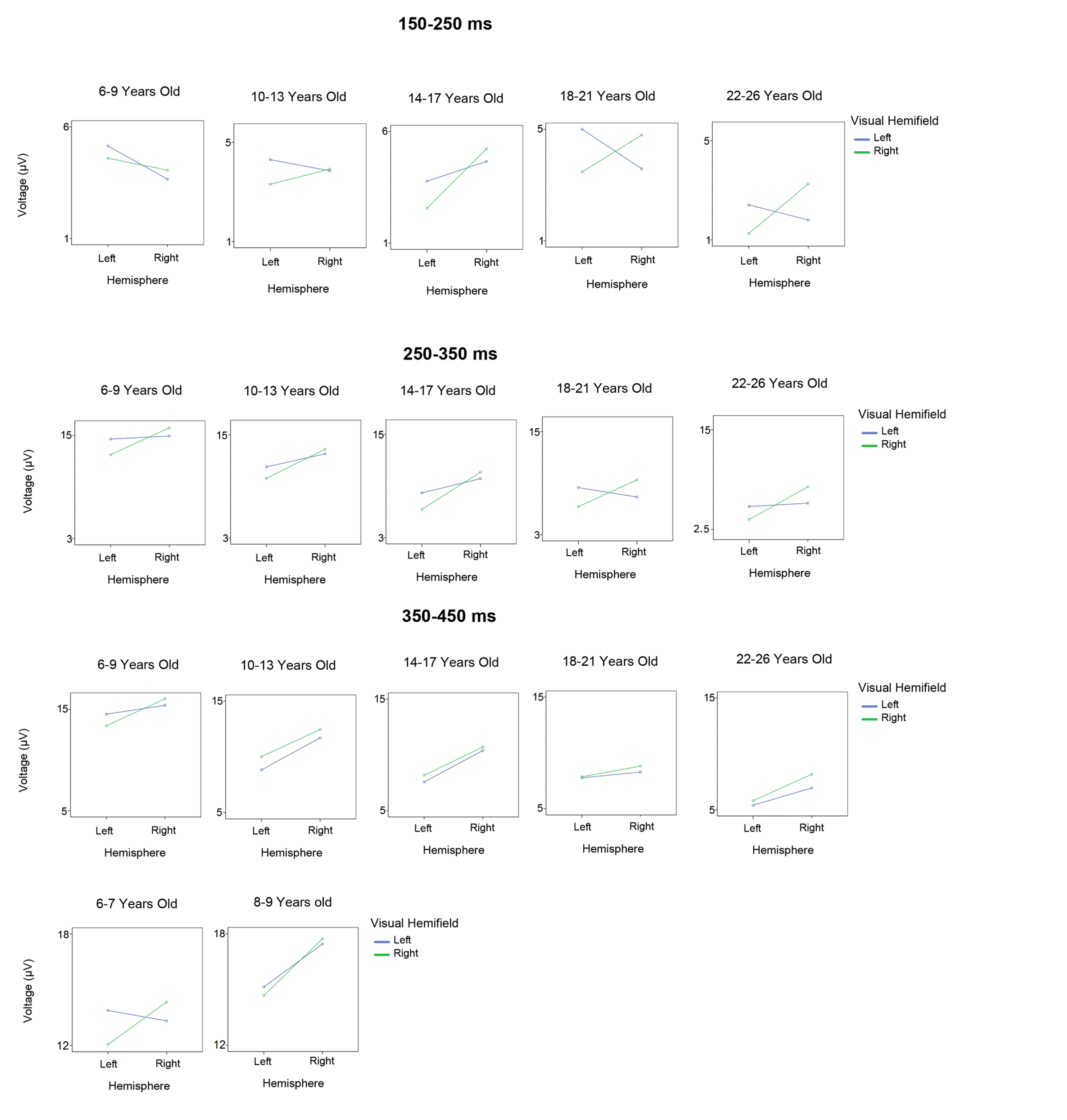

Supplement: Supplementary Figure 1 — Hemisphere × Visual Hemifield interactions in the 150–250, 250–350, and 350-450 ms for all 5 age groups. In the last time window, the interactions for the sub-groups from 6 to 7 and 8 to 9 years old are also displayed. [file Image1.TIF]
